# Halogen bonding in hypervalent iodine and bromine derivatives: halonium salts

**DOI:** 10.1107/S2052252517004262

**Published:** 2017-05-10

**Authors:** Gabriella Cavallo, Jane S. Murray, Peter Politzer, Tullio Pilati, Maurizio Ursini, Giuseppe Resnati

**Affiliations:** a NFMLab, Department of Chemistry, Materials and Chemical Engineering ‘Giulio Natta’, via Mancinelli 7, Milan I-20131, Italy; b Department of Chemistry, University of New Orleans, New Orleans, LA 70148, USA

**Keywords:** halogen bond, σ-hole interactions, hypervalent iodine, hypervalent bromine, crystal engineering

## Abstract

Halogen bonds have been identified in a series of ionic compounds involving bromonium and iodonium cations and several different anions, some also containing hypervalent atoms. The hypervalent bromine and iodine atoms in the examined compounds are found to have positive σ-holes on the extensions of their covalent bonds, while the hypervalent atoms in the anions have negative σ-holes. The positive σ-holes on the halogens of the studied halonium salts determine the linearity of the short contacts between the halogen and neutral or anionic electron donors, as usual in halogen bonds.

## Abstract 2

The presence of halogen bonds in some hypervalent iodine and bromine derivatives is demonstrated. Hypervalent atoms in polyatomic cations and anions do have σ-holes on the extensions of their covalent bonds and the cation holes are influential on the crystal lattice structures of halonium salts.

## 1. Introduction   

It is well established that the charge distribution of a covalently bonded atom is anisotropic; the electronic density on the side of the atom opposite the bond is less than on the lateral sides of the atom (Stevens, 1979 ▸; Nyburg & Wong-Ng, 1979 ▸; Price *et al.*, 1994 ▸; Awwadi *et al.*, 2006 ▸; Hathwar *et al.*, 2014 ▸; Pavan *et al.*, 2014 ▸). The region of lower electronic density along the extension of the covalent bond is called a σ-hole (Politzer *et al.*, 2013 ▸, 2015*a* ▸). Due to their lower electronic densities, σ-holes often (but not always) have positive electrostatic potentials, while the regions around them are usually negative. The atom can interact attractively through its σ-hole with negative sites, *e.g.* lone pairs, π-electrons and anions (Politzer *et al.*, 2013 ▸; Cavallo *et al.*, 2016 ▸) and through its lateral sides with positive sites, *e.g.* other σ-holes and cations (Metrangolo & Resnati, 2013 ▸; Cavallo *et al.*, 2014 ▸). The geometry of the covalent bonds around an atom thus influences the preferential directions of attractive interactions with nucleophiles and electrophiles. For instance, monovalent halogens can work as donors of electron density when interacting with hydrogen atoms or alkali and alkaline earth metal cations, and as acceptors of electron density when interacting with atoms and anions possessing lone pairs. In former cases, the electrophilic hydrogen, or the cation, enter preferentially the most negative region of the halogen atom *X*, namely the belt orthogonal to the C—*X* bond. In latter cases, the nucleophile has a preference for the σ-hole and the resulting interaction tends to form on the extension of and opposite to the C—*X* bond. In monovalent halogens attractive interactions with electrophiles and nucleophiles thus tend to be geometrically orthogonal to each other.

Interactions formed with negative sites by σ-holes on halogen atoms (Group 17) are called halogen bonds (XBs; Desiraju *et al.*, 2013 ▸) analogous to interactions wherein H atoms are the acceptors of electronic density which are named hydrogen bonds (HBs). While the first halogen-bonded adduct was reported as early as 1814 (Colin, 1814 ▸), the nature of the interaction was understood only much later, when positive σ-holes on covalently bonded halogen atoms were discovered (Brinck *et al.*, 1992 ▸, 1993 ▸). In the same years, the applicative usefulness of the interaction was fully appreciated, in conjunction with the systematization of strategies to tune the interaction strength (Metrangolo & Resnati, 2001 ▸). Halogen σ-holes become more positive, and halogen bonds stronger, as the halogen atom is more polarizable (F < Cl < Br < I) and as the remainder of the molecule is more electron-attracting (Valerio *et al.*, 2000 ▸).

**Figure 1:**
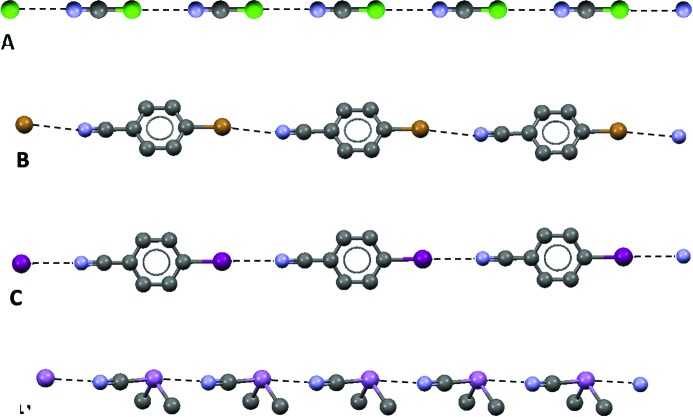
Partial representation (Mercury 3.8, ball and stick) of the infinite chains formed by halogen bonds in crystalline (*a*) cyanogen chloride, (*b*) 4-bromo- and (*c*) 4-iodobenzonitrile and formed by pnictogen bonds in (*d*) cyano-dimethylarsine. Hydrogen atoms have been omitted for simplicity; halogen and pnictogen bonds are black dotted lines. Color codes: grey, carbon; blue, nitrogen; violet, arsenic; brown, bromine; green, chlorine; purple, iodine.

Fig. 1 ▸(*a*) shows the linear halogen-bonded chain formed by cyanogen chloride in the solid state (Heiart & Carpenter, 1956 ▸) *via* C—Cl⋯N≡C halogen bonds; cyanogen bromide (Geller & Schawlow, 1955 ▸) and cyanogen iodide (Ketelaar & Zwartsenberg, 1939 ▸) behave similarly. Analogous chains are obtained with 4-bromo- and 4-iodobenzonitrile (Desiraju & Harlow, 1989 ▸; Figs. 1*b* and 1*c*), but not with the 4-chloro analogue, possibly as chlorine is a weaker XB donor.

**Figure 2:**
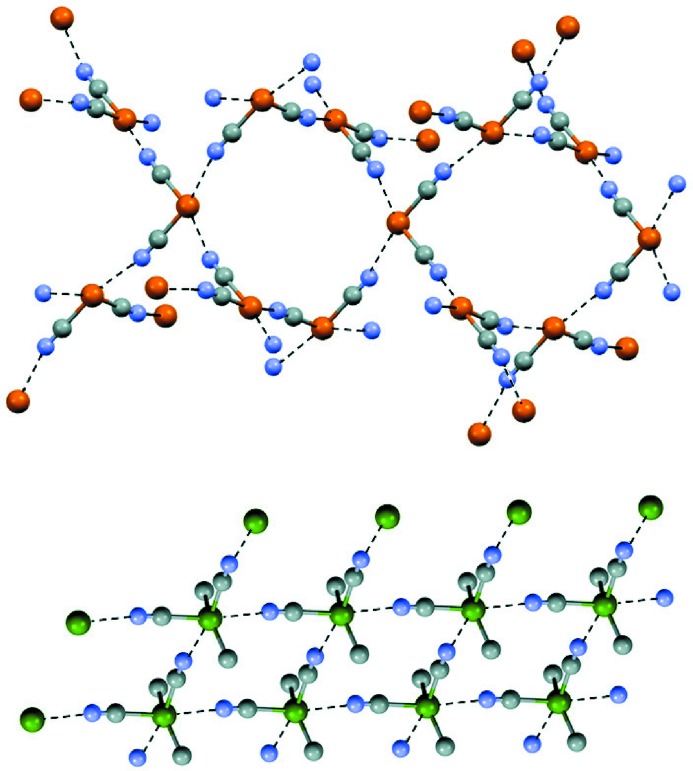
Partial representation (Mercury 3.8, ball and stick) of the two-dimensional nets formed by dicyanotelluride under chalcogen bond control (top) and by dicyano-dimethyltin under tetrel bond control (bottom). Hydrogen atoms have been omitted for simplicity; chalcogen and tetrel bonds are black dotted lines. Color codes: grey, carbon; blue, nitrogen; ochre, tellurium; dark green, tin.

The anisotropic distribution of the electronic density in an atom due to its involvement in σ-bond formation, the development of σ-holes opposite these bonds, and the directionality of interactions formed on the entrance of electrophiles in these areas of depleted electron density are not limited to monovalent halogens as they also occur with hydrogen (Murray *et al.*, 2010 ▸; Politzer *et al.*, 2013 ▸), and elements of Group 14 (Southern & Bryce, 2015 ▸), 15 (Politzer *et al.*, 2014 ▸) and 16 (Ho *et al.*, 2016 ▸). The maximum number of σ-holes on an atom, namely of directional interactions it may form with nucleophiles, is usually equal to the number of covalent bonds the atom is involved in. Also for elements of any group of the periodic table, σ-holes tend to become more positive as the molecular environment is more electron-withdrawing and as the atom is more polarizable. It should be noted that σ-holes can also have negative electrostatic potentials, especially for the less-polarizable first-row atoms, but then they are less negative than the surrounding regions. Cyanodimethylarsine produces the chain shown in Fig. 1 ▸(*d*) (Britton *et al.*, 2002 ▸) by means of the NC—As⋯N≡C pnictogen bond, while tellurium dicyanide (Klapötke *et al.*, 2004 ▸) and dicyanodimethyltin (Konnert *et al.*, 1972 ▸) form two-dimensional networks *via* the Te⋯N≡C chalcogen bond and Sn⋯N≡C tetrel bond (Fig. 2 ▸). The terms tetrel bond, pnictogen bond and chalcogen bond have been proposed to designate interactions wherein elements of Groups 14, 15 and 16 are the electrophilic site and an IUPAC Project (No. 2016-001-2-300) is pursuing a recommendation proposing a definition for these terms.

Examples reported in Fig. 2 ▸ show that elements of Groups 14 and 16, which form more than one covalent bond and have more than one σ-hole, can form more than one directional interaction with nucleophiles, and we thus reasoned that hypervalent halogens, which also form more than one covalent bond, may also have more than one σ-hole and form more than one XB. More generally, any hypervalent atom, which is involved in more than its usual number of covalent bonds, may have σ-holes on the extensions of all its bonds and may form a corresponding number of attractive interactions with sites of opposite polarity. Computationally, this has been confirmed for hypervalent atoms in a number of neutral molecules (Clark *et al.*, 2008 ▸; Murray *et al.*, 2009 ▸; O’Hair *et al.*, 2010 ▸). We have now investigated, both computationally and experimentally, a series of ionic compounds in which the cation and sometimes the anion contain hypervalent atoms. Specifically, we have established the surface electrostatic potential of the cation and anion of some ionic λ^3^-iodane and λ^3^-bromane derivatives, and we have checked the contacts below the sum of the van der Waals radii of involved atoms (hereinafter named short contacts) formed by these halogens in the crystalline state. It is known that the electrostatic potential of a cation (anion) is positive (negative) everywhere on its surface, although the values of the electrostatic potential cover ranges of positive (negative) values except on the surfaces of monoatomic cations (anions) (Politzer *et al.*, 2016 ▸). Accordingly the hypervalent atoms will be positive or negative on their entire surfaces in the cation or anion, respectively, but are these atoms more positive (in the case of cations) or less negative (in the case of anions) on the extensions of the covalent bonds to them, thus forming σ-holes? Moreover, are the positive σ-holes on halogens of hypervalent halonium cations influential in the formation of short contacts with anions in the solid? In this paper we show that answers to these questions are yes for the salts of ionic λ^3^-iodane and λ^3^-bromane, and the directionality of the short contacts involving halogen atoms in these hypervalent iodine and bromine compounds fulfill the prerequisites for being considered bona fide XBs. The short contacts involving hypervalent halogens are frequently named in the literature as secondary bondings (Alcock & Countryman, 1977 ▸); an advantage of naming them halogen bonds is that this terminology is more descriptive of some of the interaction features; for instance, it immediately gives indications of the directionality of the interaction and the relationships between the strength of the interaction and the nature of involved sites (Catalano *et al.*, 2016 ▸).

## 2. Experimental   

### 2.1. Materials and methods   

Phenyl-2-carbomethoxyphenyl-bromonium tetrafluoroborate, dibenzo[*b*,*d*]iodolium chloride, diphenyliodonium perchlorate, diphenyliodonium hexafluorophosphate and di-*p*-fluorophenylbromonium tetrafluoroborate were purchased from TCI or Vitas-M Laboratory, LTD. Di-*p*-fluorophenylbromonium chloride and bromide were prepared starting from the corresponding tetrafluoroborate salt by using anion exchange resins, Amberlite IRA 900 (Cl^−^ content: 4.2 mmol g^−1^) and Amberlyst A-26 Br^−^ form (Br^−^ content: 3.5 mmol g^−1^), respectively. The starting tetrafluoroborate salt (3.0 mmol) was dissolved in methanol (70 ml) and the solution was left in contact with the resin (enough resin was used in order to have a 3:1 halide/tetrafluoroborate ratio). After 20 h at room temperature, the resin was filtered and substituted with fresh resin four times. On evaporation of the final methanol solution, **4·Cl^−^** and **4·Br^−^** were obtained in pure form; the content of residual **4·BF_4_^−^** was established < 0.05% in weight through ^19^F NMR after addition of **4·BF_4_^−^** as an internal standard.

### 2.2. X-ray structure analyses   

Good quality single crystals of all iodonium and bromonium derivatives were grown by slow solvent evaporation techniques under isothermal conditions at 298 K. In a typical crystallization procedure, a saturated solution of the halonium salt(s) in methanol (in order to obtain **1·ClO_4_^−^**, **1·PF_6_^−^**, **3·BF_4_^−^**, **4_3_·(Br^−^)_2_·BF_4_^−^** and **4_3_·(Cl^−^)_2_·BF_4_^−^**) or hexafluoroisopropanol (in order to obtain **2·Cl^−^**) was prepared at room temperature in a clear borosilicate glass vial which was left open in a closed cylindrical wide-mouth bottle containing paraffin oil. Solvents were allowed to slowly evaporate at room temperature and to be absorbed by paraffin oil until crystals were formed in a period ranging 3–5 days.

All the crystal data were collected with a Bruker APEX2000 diffractometer, with Mo *K*α radiation, λ = 0.71073 Å, and collected at 103 K with the temperature controlled by a Bruker KRYOFLEX device. Data reduction and empirical absorption correction were carried out using *SAINT* and *SADABS* (Sheldrick, 1996 ▸). The structures were solved by direct methods (*SIR*2002) and refined on *F* ^2^ by *SHELXL97*. Tables S1 and S2 of the supporting information report the main data of the crystal structures containing cations **1**–**4**. CCDC Nos. 1532402–1532407 contain the supplementary crystallographic data. These data can be obtained free of charge from the Cambridge Crystallographic Data Centre *via* http:www.ccdc.cam.ac.uk/data_request/cif.

## 3. Results and discussion   

**Figure 3:**
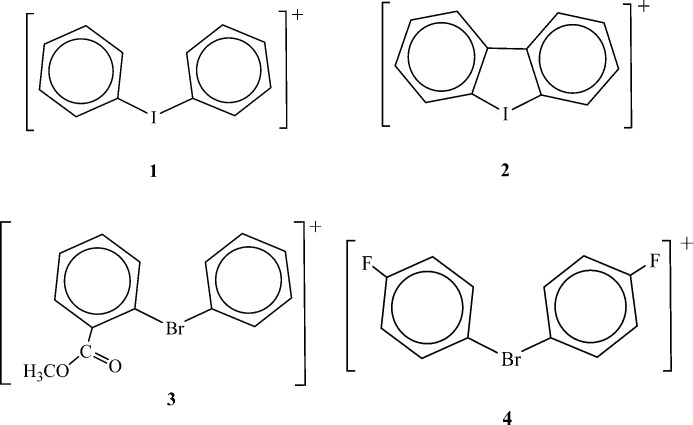
Structures of studied iodonium and bromonium cations.

The cations upon which we will focus involve hypervalent bromines and hypervalent iodines. Each halogen atom is bonded covalently to two aryl groups, forming diarylbromonium and diaryliodonium cations. Derivatives of these are quite well known (Merritt & Olofsson, 2009 ▸). We will consider the following cations: diphenyliodonium (**1**), dibenzo[*b*,*d*]iodonium (**2**), phenyl-2-methoxycarbonylphenylbromonium (**3**) and di-*p*-fluorophenylbromonium (**4**) (Fig. 3 ▸). The anions include chloride (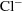), bromide (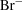), perchlorate (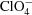), tetrafluoroborate (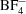) and hexafluorophosphate (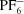). The last three anions contain hypervalent chlorine, boron and phosphorus, respectively.

### 3.1. Computational modelling   

To determine whether the hypervalent atoms in the cations and anions of interest do have σ-holes on the extensions of the covalent bonds to these atoms, we have computed optimized geometries using *GAUSSIAN09* (Frisch *et al.*, 2009 ▸) and electrostatic potentials on the surfaces of the cations **1–4**, as well as the anions that contain hypervalent atoms: 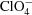, 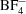 and 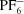. Frequency calculations were performed for each cation and anion to confirm the absence of imaginary frequencies. (The monatomic 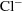 and 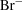 anions have uniform negative potentials over their spherical surfaces.) The ionic surfaces were taken to be the 0.001 a.u. contours of the cations’ or anions’ electronic densities (Bader *et al.*, 1987 ▸) and the WFA-SAS (wave function analysis-surface analysis suite) code was used to obtain the electrostatic potentials (Bulat *et al.*, 2010 ▸). The computational procedure was the M06-2X/6-311G(d). The M06-2X is a hybrid meta density functional developed for treating noncovalent interactions (Zhao & Truhlar, 2008 ▸), while the 6-311G(d) basis set has been found to be effective for molecular surface electrostatic potentials (Riley *et al.*, 2016 ▸), including systems containing the larger halogens iodine and bromine (Riley *et al.*, 2011 ▸).

**Figure 4:**
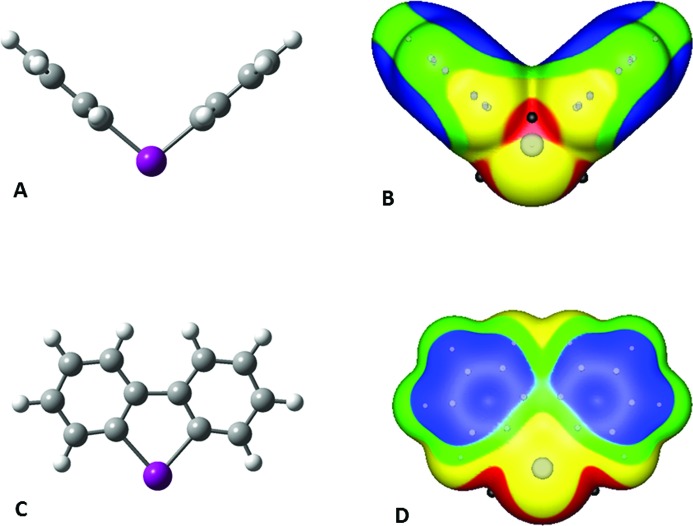
Calculated ball-and-stick structure of (*a*) diphenyliodonium cation (**1**) and of (*c*) dibenzo[*b*,*d*]iodonium cation (**2**). Color code: gray, carbon; white, hydrogen; purple, iodine. Computed electrostatic potential on the 0.001 a.u. surface of cations **1** (*b*) and **2** (*d*) showing the same view as in (*a*) and (*c*), respectively. Color ranges, in kcal mol^−1^: red, greater than 100; yellow, between 100 and 85; green, between 85 and 75; blue, less than 75. The black hemispheres indicate the most positive potentials of the iodine σ-holes on the extensions of the C—I bonds; the black sphere in (*b*) at the center corresponds to another potential maximum.

The calculated structure and the surface electrostatic potential of the cation **1** is shown in Figs. 4 ▸(*a*) and (*b*). **1** has a V-shaped geometry, with a C—I^+^—C angle of about 99° between the two phenyl rings. The potential on the surface is positive everywhere, as is expected for a cation, but the values of the electrostatic potential varies considerably. The most positive regions are on the extensions of the C—I bonds, with maximum values of 111 kcal mol^−1^. These correspond to σ-holes on the iodine. There are also strongly positive potentials in the cleaves between the two phenyl rings on both sides of **1**, with maxima of 106 kcal mol^−1^. However, they are much less accessible to interactions with negative sites than are the two σ-holes on the iodine.

The least positive potentials on the surface of **1** are approximately 60 kcal mol^−1^. They are associated with the π regions of the phenyl rings.

The bromine analogue of **1**, the diphenylbromonium cation (not shown), has a structure and surface potential very similar to those of **1**. However, the most positive potentials of the two σ-holes, on the extensions of the C—Br bonds, are 108 kcal mol^−1^, slightly less than the σ-holes of **1**. This is consistent with the lesser polarizability of bromine compared with iodine.

Cations **1** and **2** differ in that **2** has a C—C bond linking the two phenyl rings. This has a major effect upon its structure, which is planar (Fig. 4 ▸ *c*). The C—I^+^—C angle is 82°. However, the surface electrostatic potential of **2** (Fig. 4 ▸ *d*) again has its most positive regions on the extensions of the C—I bonds, denoting σ-holes on the iodine; their maximum values are 127 kcal mol^−1^.

**Figure 5:**
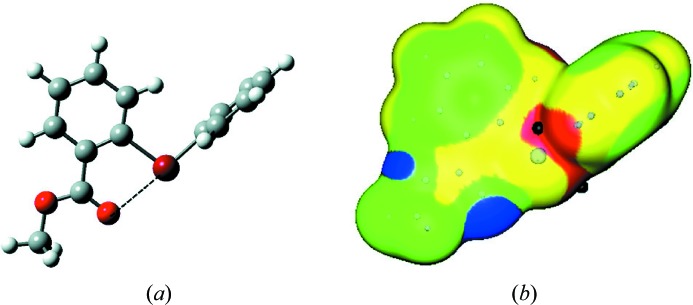
(*a*) Calculated ball-and-stick structure of the phenyl-2-methoxycarbonylphenyl-bromonium cation, **3**. Color code: gray, carbon; white, hydrogen; bright red, oxygen; dark red, bromine. The methoxy­carbonyl group is at the bottom. The dashed line indicates the intra-ionic 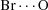 interaction. (*b*) Computed electrostatic potential on the 0.001 a.u. surface of cation **3**, showing the same view as in (*a*). Color ranges, in kcal mol^−1^: red, greater than 90; yellow, between 90 and 75; green, between 75 and 60; blue, less than 60. The black hemisphere indicates the most positive potential of the lone σ-hole on the bromine, on the extension of the C—Br bond; the black sphere in the center corresponds to another potential maximum.

Cation **3** has an interesting structural feature, which can be seen in Fig. 5 ▸(*a*). While the computed C—Br^+^—C angle of approximately 101° is similar to the C—I^+^—C angle of 99° in **1**, the ring that bears the methoxycarbonyl group in **3** is predicted to be considerably rotated. This can be attributed to an attractive interaction (dashed line) between the carbonyl oxygen and the nearby σ-hole of the bromine. The calculated Br⋯O separation is 2.628 Å and the C—Br⋯O angle is nearly linear at 176.9°. This is an intra-ionic XB.

A close contact is sometimes characterized by means of the corresponding ‘normalized contact’, Nc. This is defined as the ratio of the observed or calculated separation to the sum of the respective van der Waals atomic radii or Pauling ionic radii. Nc < 1 is taken to indicate an attractive interaction. The computed 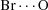 distance in **3** gives an Nc of 0.75. Intra-ionic 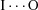 bonds have also been observed, with Nc of 0.73, in substituted diphenyliodonium cations (Halton *et al.*, 2001 ▸; Zhdankin *et al.*, 2003 ▸).

The electrostatic potential on the surface of **3** (Fig. 5 ▸ *B*) reflects the rotation and intra-ionic interaction that have been described. The bromine does have a σ-hole, on the extension of the C—Br bond from the rotated ring; its maximum potential is 101 kcal mol^−1^. However, the other σ-hole that would be expected for this hypervalent bromine is involved in the interaction with the carbonyl oxygen and therefore is not visible. The cation **3** also has strongly positive potentials in the cleaves between the two rings on both sides of **3**, analogous to what is in **1**. Their largest values are 97 kcal mol^−1^, but they are rather poorly accessible.

It is interesting that an attractive interaction does occur in **3** between a positive σ-hole and the carbonyl oxygen, since the latter is also positive, although less so than other portions of the cationic surface (Fig. 5 ▸ *b*). This may be due to the polarization induced by the strongly positive potential of the σ-hole with which the oxygen is interacting. This draws electronic charge to the oxygen and makes it less positive. This intra-ionic attractive interaction can be viewed as noncovalent (Politzer *et al.*, 2015*b* ▸). Analogous effects have been observed in the past (Clark *et al.*, 2014 ▸; Politzer *et al.*, 2015*a* ▸).

**Figure 6:**
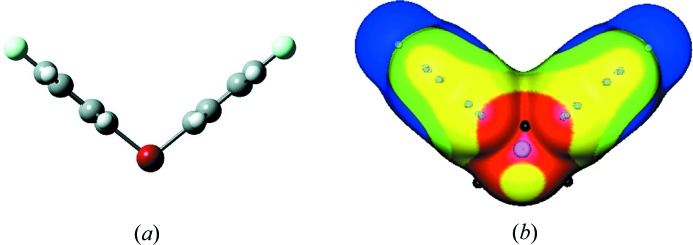
(*a*) Calculated ball-and-stick structure of the di-*p*-fluorophenylbromonium cation, **4**. Color code: gray, carbon; white, hydrogen; light blue, fluorine; dark red, bromine. (*b*) Computed electrostatic potential on the 0.001 a.u. surface of cation **4**, showing the same view as in (*a*). Color ranges, in kcal mol^−1^: red, greater than 100; yellow, between 100 and 85; green, between 85 and 75; blue, less than 75. The black hemispheres indicate the most positive potentials of the bromine σ-holes, on the extensions of the C—Br bonds; the black sphere in the center corresponds to another potential maximum.

Cation **4** has a V-shaped geometry (Fig. 6 ▸ *a*), similar to that of **1**. The C—Br^+^—C angle is 102°. There are σ-holes on the extensions of the C—Br bonds, with maximum values of 114 kcal mol^−1^ (Fig. 6 ▸ *b*). As in **1** and **3**, there are also strongly positive regions in the cleaves between the rings, on both sides of **4**. Their most positive potentials are 116 kcal mol^−1^, slightly higher than those of the σ-holes on the bromine. However, these positive regions are buried within the cleaves, whereas those of the bromine are on the outside, easily accessible. The fact that the σ-hole maxima in **4** are greater than the 111 kcal mol^−1^ in **1**, even though iodine is more polarizable than bromine, is due to the electron-attracting fluorines in **4**. The fluorines, in turn, are the least positive portions of **4**. Their lowest potentials are 45 kcal mol^−1^.

**Figure 7:**
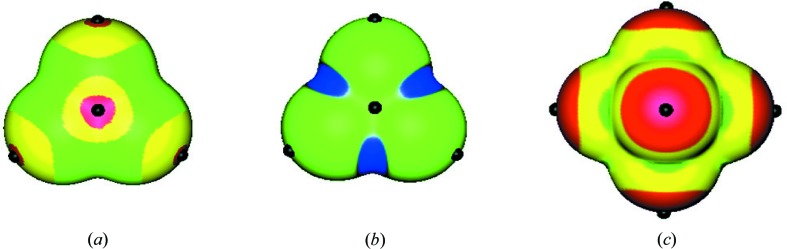
Computed electrostatic potentials on the 0.001 a.u. surfaces of (*a*) 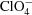, (*b*) 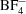 and (*c*) 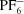. Color ranges, in kcal mol^−1^: red, less negative than −115; yellow, between −115 and −120; green, between −120 and −133; blue, more negative than −133. In (*a*) and (*b*), the black hemispheres and sphere indicate the least negative potentials of the σ-holes of the peripheral atoms and the central atom; in (*c*) they indicate the least negative potentials of the σ-holes of just the peripheral atoms.

The electrostatic potentials on the surfaces of the anions 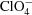, 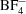 and 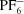 are displayed in Fig. 7 ▸. The two tetrahedral ones, 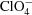 and 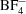, each have eight σ-holes. Four are on the peripheral O atoms or fluorines, on the extensions of the Cl—O or B—F bonds; another four are on the chlorine or boron, on the extensions of the same bonds, Cl—O or B—F, respectively. All of these σ-holes have negative potentials, of course, but they are less negative than the surrounding regions. The most negative potentials on the surfaces of these two anions are between the peripheral atoms.

The 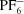 anion presents an interesting feature: There are no visible σ-holes on the phosphorus. This is the result of the octahedral symmetry of the anion; on the extension of each F—P bond is another F—P bond; these block each other’s potential σ-holes on the phosphorus. The only visible σ-holes on this anion’s surface are on the fluorines, on the extensions of the P—F bonds. The most negative regions are between the fluorines.

Figs. 3–7 ▸ ▸ ▸ ▸ ▸ confirm that hypervalent atoms in polyatomic cations and anions do have σ-holes on the extensions of their covalent bonds, unless there is some special circumstance such as the intra-ionic interaction in **3** or the octahedral symmetry of 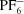. The σ-holes are more positive than the regions around them for the cations, and less negative than the surrounding regions for the anions.

### 3.2. Structural studies   

In all examined structures HBs between aromatic H atoms and anions are present, consistent with the calculated positive electrostatic potential at H atoms of aryl halonium derivatives. However, the intermolecular interactions showing the lowest Nc values are, in all structures, the two most linear XBs between iodine, or bromine, atoms and anions, or lone-pair possessing atoms. The focus of this section will be on these short contacts.

**Figure 8:**
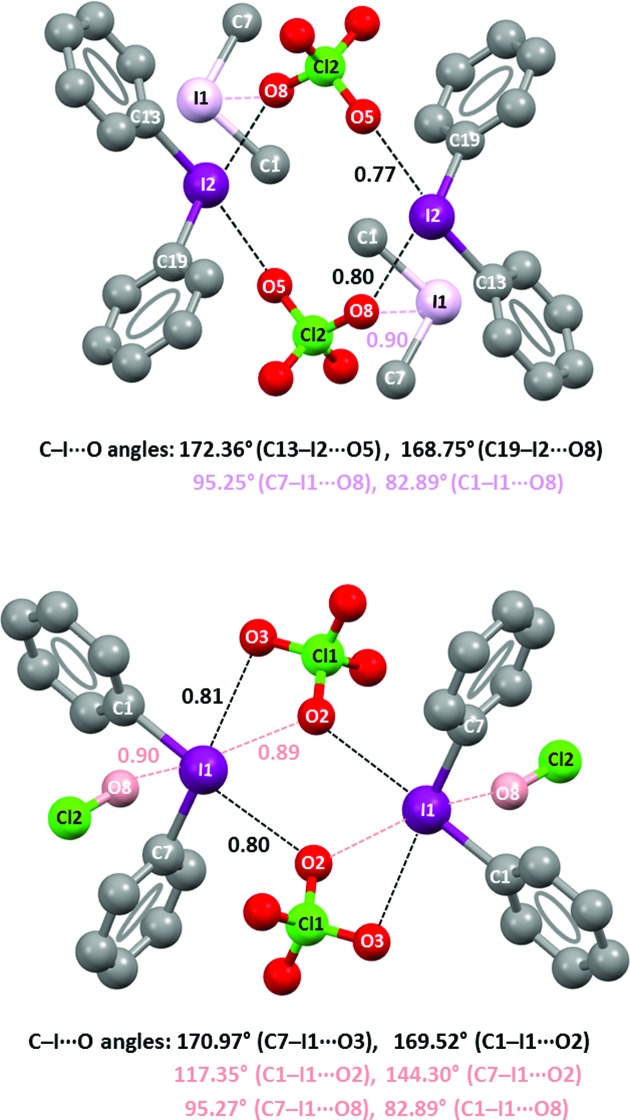
Representation (Mercury 3.8, ball and stick) of the two different tetrameric adducts formed by the I⋯O XBs in the crystal lattice of **1·ClO_4_^−^**. Hydrogen atoms have been omitted for simplicity; Nc values of XBs and angles are given close to interactions and at the bottom, respectively. Color code: grey, carbon; red, oxygen; green, chlorine; purple, iodine. In the top representation I1, the I⋯O short contact it forms out of C—I bond elongations and corresponding parameters are in pink. In the bottom representation O8, the I⋯O short contact it forms out of C—I bond elongations and corresponding parameters are in orange; also I⋯O XB formed by O2 out of C—I bond elongations and corresponding parameters are in orange.

Diphenyliodonium perchlorate (**1·ClO_4_^−^**) forms, in the crystal lattice, tetrameric adducts which are assembled thanks to short 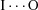 XBs. The asymmetric unit of the crystal contains two independent cation–anion couples which form two different tetrameric adducts around two inversion centers. Topologically speaking, these tetramers are parallelograms wherein two iodine atoms and two 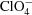 anions are the vertexes and two pairs of 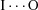 contacts are the sides. Perchlorate anions function as bidentate XB acceptors, two oxygen atoms of the two independent perchlorate anions enter iodonium sites along the prolongation of the two C—I bonds and form short and directional 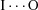 contacts (black dashed lines in Fig. 8 ▸). These 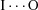 separations are 2.8761 (10) and 3.0089 (12) Å long in one tetramer (Fig. 8 ▸, top) and 2.9153 (9) and 3.0147 (11) Å in the other (Fig. 8 ▸, bottom), these values corresponding to Nc in the range 0.77–0.81. The respective C—I⋯O angles are 172.36 (4)° and 168.75 (4)° for the former tetramer and 169.52 (3)° and 170.97 (3)° for the latter. Other I⋯O short contacts are present (pink and orange dashed lines in Fig. 8 ▸), but they are longer and less linear than those described above (the average I⋯O separation is 3.340 Å (Nc = 0.90), and the average C—I⋯O angle is 103°). These structural features are consistent with the calculated anisotropic distribution of the electron density on iodine where two σ-holes are opposite to the C—I covalent bonds: When anions enter these holes, the resulting I⋯O contacts are shorter than when entering far from the holes. The overall pattern of interactions can be understood as charge-assisted and bifurcated XBs. Cation–anion attraction plays a role in I⋯O short contacts (*i.e.* they are charge-assisted interactions) and the σ-hole presence accounts for why they are shorter when the C—I⋯O angles are close to linearity.

**Figure 9:**
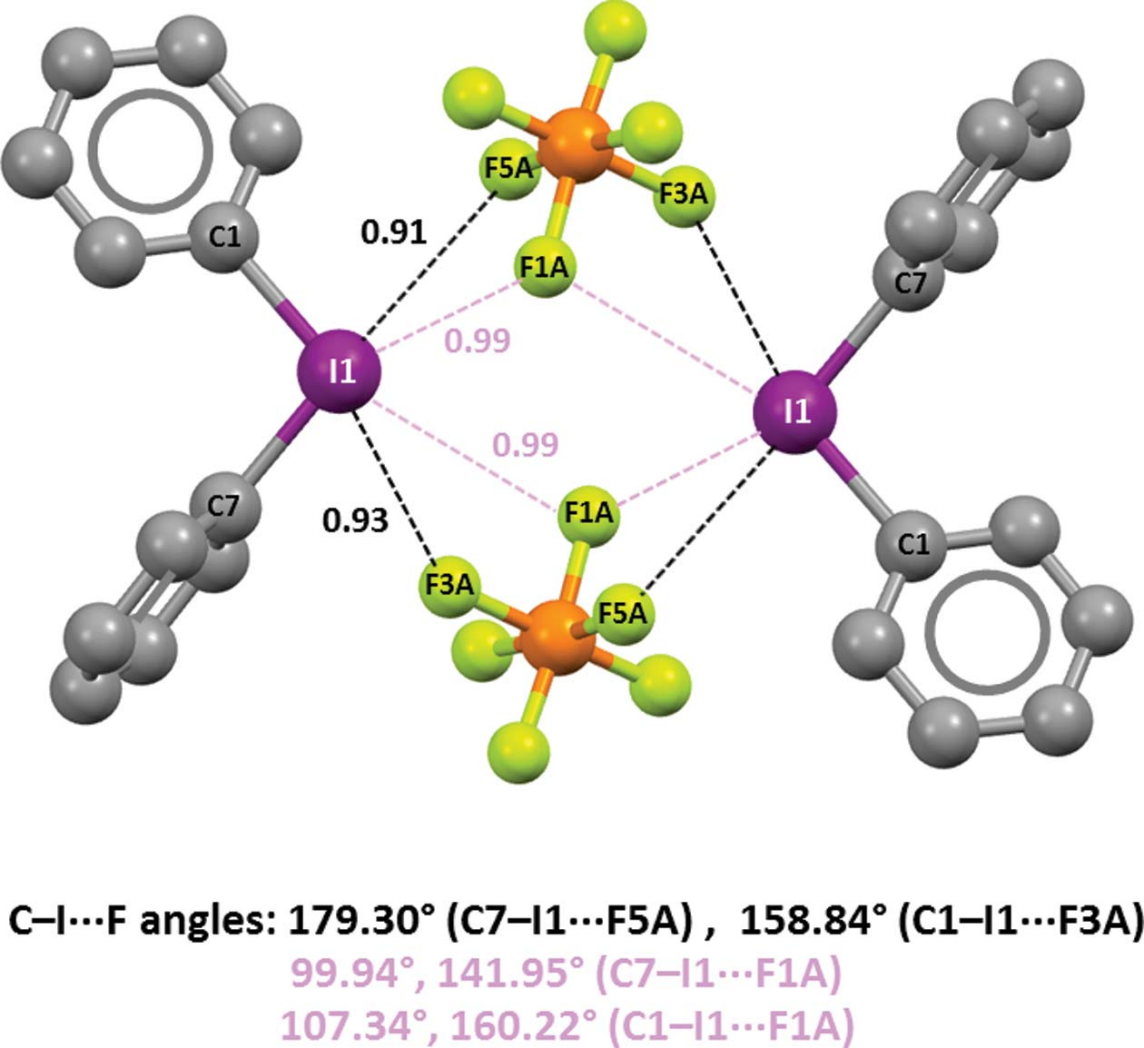
Representation (Mercury 3.8, ball and stick) of the tetrameric adduct formed by the I⋯F XBs in the crystal lattice of **1·PF_6_^−^**. Hydrogen atoms have been omitted for simplicity; Nc values of XBs and angles are given close to interactions and at the bottom, respectively. Color code: grey, carbon; yellowish green, fluorine; purple, iodine; orange, phosphorus. Less short I⋯F contacts and corresponding parameters are in pink.

Bifurcated XBs are attractive interactions wherein two electron-rich sites enter a σ-hole on a halogen, after either symmetric or dissymmetric geometry (Ji *et al.*, 2011 ▸). Bifurcated XBs are rarely formed by monovalent halocarbons, possibly as the surface electrostatic potential in these compounds typically changes from positive at the hole to negative at the orthogonal belt. The electrostatic potential remains positive on the whole surface of the halogens in iodonium, and bromonium, derivatives and it may be expected that bifurcated XBs are not as rare for these donors. Diphenyliodonium hexafluorophosphate (**1·PF_6_^−^**) forms tetrameric adducts similar to those in **1·ClO_4_^−^**. Two 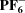 units are pinned in their position thanks to two fluorine atoms (F3A and F5A) entering C—I bonds extensions (C1—I1 and C7—I1, respectively) and forming short I⋯F contacts (Nc = 0.91 and 0.93, black dashed lines in Fig. 9 ▸). Here too bifurcated XBs are present; in fact, the covalent connectivity within 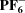 delivers a third fluorine (F1A) close to the iodine atoms. Further I⋯F XBs are formed, but they are much longer than the XBs discussed above (Nc = 0.99, pink dashed lines in Fig. 9 ▸).

Crystalline **1·PF_6_^−^** provides a good example of how the anion can influence the structure of the cation. In fact, in the lattice the cation does not have the equilibrium geometry of the free cation **1** (Fig. 4 ▸), possibly due to the presence of a net of attractive H⋯F hydrogen bonds which pin the phenyl rings in their position. Additionally in **1·ClO_4_^−^** the cation does not have the equilibrium geometry and the net of H⋯O hydrogen bonds may also be the cause in that case.

Dibenzo[*b*,*d*]iodolium chloride (**2·ClO^−^**) is a cell-permeable, irreversible inhibitor of endothelial nitric oxide synthase (Xu *et al.*, 2014 ▸) and in the solid it forms discrete tetrameric adducts (Fig. S1) *via* linear 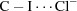 interactions. Each chloride and each iodine is involved in two such interactions and alternates at the vertexes of a parallelogram whose sides, the I⋯Cl^−^ interactions, are in the range 3.0694 (9)–3.1781 (6) Å (Nc being 0.81–0.83). The C—I⋯Cl^−1^ angles vary between 170.32 (5)° and 174.70 (5)°. C—I⋯Cl^−^ interactions are clearly XBs along the extensions of the C—I bonds and between the iodine σ-holes depicted in Fig. 4 ▸ and the chloride ions.

**Figure 10:**
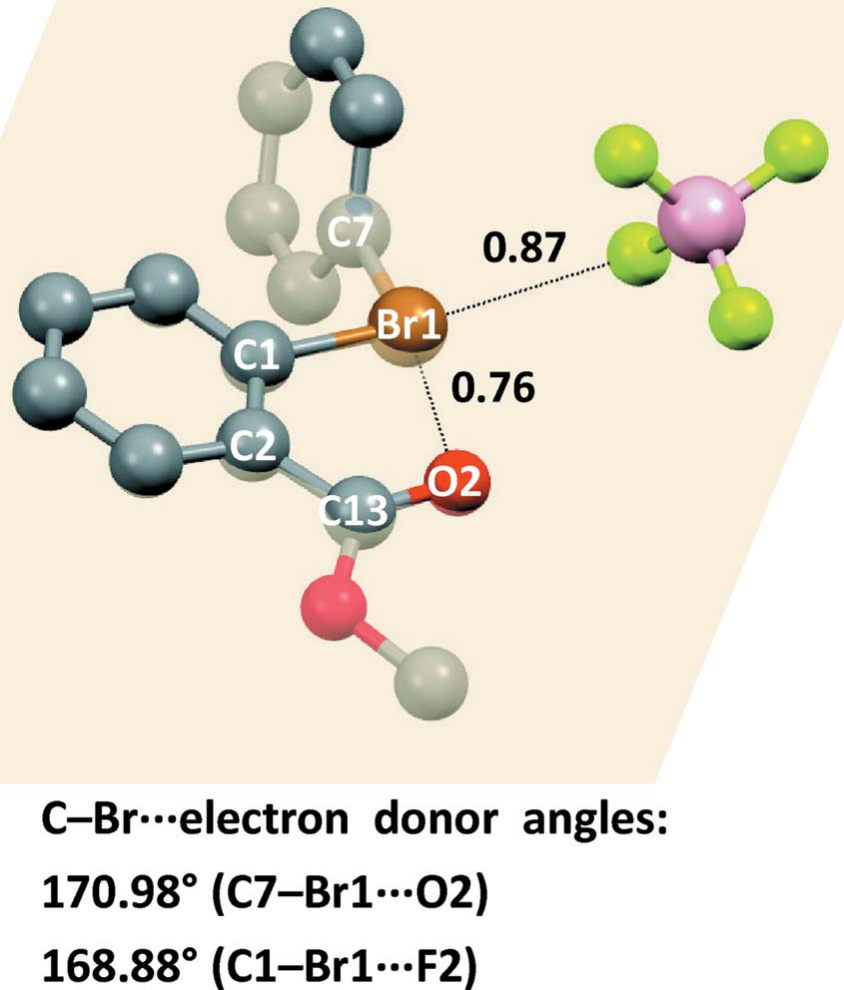
Representation (Mercury 3.8, ball and stick) of diphenyl bromonium derivative **3·BF_4_^−^**. The mean square plane through Br1, C1, C2, C13 and O2 (semitransparent yellowish) shows that fluorine and oxygen atoms enter the elongation of C—Br covalent bonds. Hydrogen atoms have been omitted for simplicity; XBs are black dotted lines, the respective Nc values and angles are given close to interactions and at the bottom, in order. Color codes (above mean square plane): grey, carbon; red, oxygen; brown, bromine; yellowish green, fluorine, pink, boron.

The crystal lattice of phenyl-2-methoxycarbonylphenylbromonium tetrafluoroborate (**3·BF_4_^−^**) shows that the intra-ionic Br⋯O halogen bond that was found computationally in the free cation **3**, between the carbonyl oxygen and the nearest bromine σ-hole, remains intact in the tetrafluoroborate salt. The Br⋯O separation observed crystallographically in the salt is 2.6476 (12) Å, with Nc = 0.76; these are close to the calculated values for the free cation, 2.628 Å and Nc = 0.75. The experimental C—Br⋯O angle is 170.98 (6)° in the crystal *versus* 176.9 (6)° in the computed free cation. The presence of the tetrafluoroborate anion does not disrupt the Br⋯O XB in the cation, probably as this is an intramolecular interaction forming a five-membered ring (Fig. 10 ▸).

Similar intramolecular I⋯O contacts (2.615 and 2.638 Å long, Nc = 0.73) are observed in a phenyl-2-carboxamidophenyliodonium trifluoromethanesulfonate and in a phenyl-2-acylphenyl iodonium trifluoromethanesulfonate (Zhdankin *et al.*, 2003 ▸; Halton *et al.*, 2001 ▸) where the carbonyl oxygen gets close to the positive halogen on the elongation of the C—I bond. Analogous arrangements are observed in phenyl-alkenyl iodonium trifluoromethanesulfonates bearing a conveniently positioned carbonyl (Williamson *et al.*, 1993 ▸). It is interesting to observe that a neutral donor of electron density can prevail over poorly nucleophilic anions in entering bromonium or iodonium sites not only when intra- but also intermolecular interactions are formed (Ochiai *et al.*, 2003 ▸; Suefuji *et al.*, 2006 ▸).

The other σ-hole on the bromine in **3·BF_4_^−^** interacts with a fluorine atom of the anion (Fig. 10 ▸). The Br⋯F distance is 2.8900 (12) Å, which yields Nc = 0.87, and the C—Br⋯F angle is 168.88 (5)°. This nearly linear arrangement of atoms in the interaction is consistent with the computed anisotropic distribution of the electron density on bromine and with the two σ-holes opposite the C—Br covalent bonds.

Finally we crystallized solutions containing equimolar amounts of di-*p*-fluorophenylbromonium tetrafluoroborate and di-*p*-fluorophenylbromonium chloride, or bromide. We examined initially formed crystals (precipitation of 10–15% of starting materials). The objective was to determine whether (*a*) different crystals containing a single anion were formed (and if so, what was the ratio of the two different crystals), or (*b*) the precipitation of a mixed cocrystal containing both anions was preferred (and if so, what was the composition).

**Figure 11:**
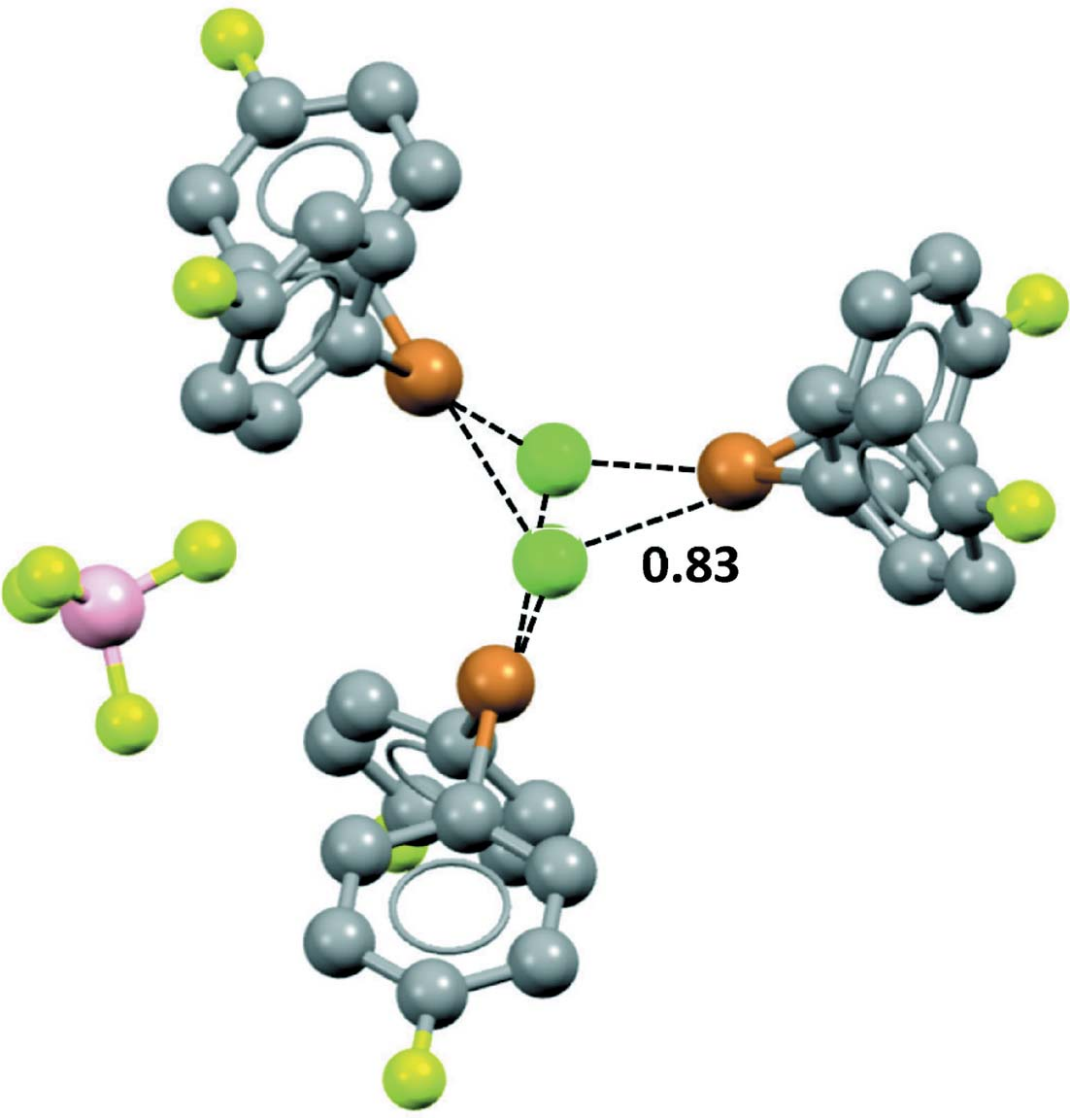
Representation (Mercury 3.8, ball and stick) of the trigonal bipyramidal adduct present in **4_3_·(Cl^−^)_2_·BF_4_^−^**. Br⋯Cl^−^ XBs are black dashed lines and corresponding Nc values are given; hydrogen atoms have been omitted for simplicity. Color code: grey, carbon; yellowish green, fluorine; pink, boron; brown, bromine; green, chlorine.

The fluoborate/chloride and fluoborate/bromide mixtures behave similarly. DSC analyses showed that both mixtures afford reproducibly a single crystalline species and X-ray analyses revealed that crystals formed by the chloride and bromide mixtures are both containing the halide and fluoborate anions in 2:1 ratio (they are thus denoted **4_3_·(Cl^−^)_2_·BF_4_^−^** and **4_3_·(Br^−^)_2_·BF_4_^−^**). In the crystal lattices are trigonal bipyramidal adducts in which the bromine of each cation **4** has short contacts with two chloride ions (Fig. 11 ▸), or two bromide ions (Fig. S2), along the extensions of the C—Br bonds. Each halide ion, in turn, interacts with three different bromine σ-holes. These are all C—Br---Cl^−^ or C—Br---Br^−1^ halogen bonds; the interactions are nearly linear, with the C—Br---Cl^−^ and C—Br---Br^−^ angles being 173.40 (5)° and 174.18 (11)°, respectively. The Br---Cl^−^ separations are 3.0493 (5) Å, the Br---Cl^−^ are 3.1595 (6) Å. Nc is 0.83 in both cases.

The chloride and bromide anions also form hydrogen bonds with the phenyl rings, as do the fluorines of the tetrafluoroborate anions. However, the latter anions do not interact with the σ-holes of the bromines; the 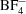 fluorines do not enter into Br---F halogen bonds, as they do in the lattice structure shown in Fig. 10 ▸. The bromine σ-holes interact preferentially with the chloride and bromide anions. Monovalent halogens show the same preference: They are more prone to halogen bonds with chloride and bromide anions than with the fluorines of the tetrafluoroborate anion (Metrangolo *et al.*, 2009 ▸).

The C—Br---Br^−^ interactions in crystalline 4-bromopyridium bromide have lower Nc values, 0.95 (Freytag *et al.*, 1999 ▸), than the C—Br---F in crystalline 4-bromopyridium tetrafluoroborate, Nc = 0.99 (Awwadi *et al.*, 2012 ▸), which may be interpreted as suggesting that the former interactions are stronger. The preference for the monatomic anions can probably be attributed to their negative charges being more concentrated than that of the polyatomic 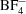 anion.

## 4. Conclusions   

We have demonstrated that hypervalent atoms that are constituents of polyatomic cations and anions do have a σ-hole on the extension of each covalent bond, unless there is some complicating factor such as the intra-ionic interaction in the cation **3** or the octahedral symmetry in the 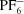 anion. In the anions, the σ-holes are less negative than the surrounding region and in the cations they are more positive than their surroundings.

The positive σ-holes on the cations can interact attractively with negative sites, as in the lattice of bromonion and iodonium derivatives shown in Figs. 8–11 ▸ ▸ ▸ ▸. The negative σ-holes on the anions can similarly interact with positive sites, and it must be recognized that positive sites may interact preferentially with the more negative regions around the negative σ-holes.

**Figure 12:**
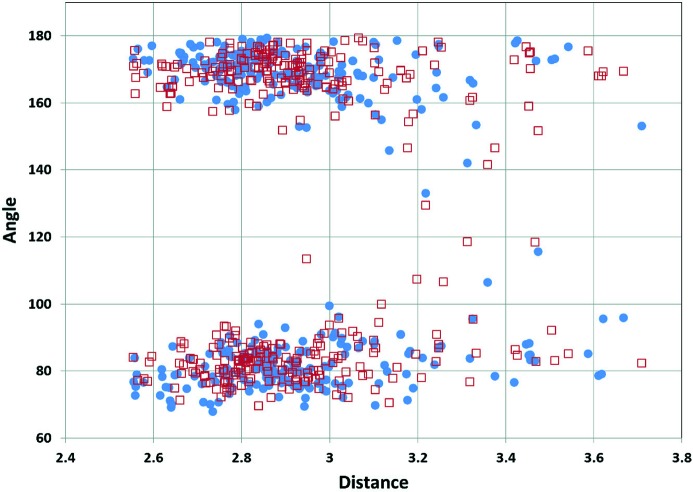
Distribution of the two C—I^+^⋯electron donor angles (°) *versus* XB distances for CCDC structures containing the C—I^+^—C moiety. The analysis has been made using the Cambridge Structure Database (CSD, Version 5.37, November 2015, including updates until February 2016). Blue dots and red squares are the two angles associated with any XB; for bi-, trifurcated XBs, the most linear was selected. The mean value of the C—I^+^—C angle in the considered structures is 94.08°; the clustering of the C—I^+^⋯electron donor angles around 90° and 170° is indicative of their preferential linearity.

In this context, we emphasize that interpretations concerning attractive interactions within ionic lattices should be made especially cautiously. Crystallographic analyses can reveal close contacts, and computed electrostatic potentials provide insight into the variations of the positive potentials on cationic surfaces and the negative potentials on anionic ones. These variations account for the directional preferences in the XBs between the halogen atom of iodonium and bromonium cations **1–4** and neutral and anionic sites. This preferential directionality is a common feature of halonium derivatives and Fig. 12 ▸ shows it for iodonium structures in the Cambridge Crystallographic Data Centre (CCDC).

It must also be kept in mind that the cations are completely positive and anions completely negative; thus any portion of one will interact attractively with any portion of the other, the strength of the interaction depending upon the separation. This feature may play a major role in the occurrence of the bifurcated halogen bonds depicted in Figs. 8 ▸ and 9 ▸.

## Supplementary Material

Crystal structure: contains datablock(s) gc131, gc140LT, gc143, gc148, gc154, gc162. DOI: 10.1107/S2052252517004262/lc5079sup1.cif

Structure factors: contains datablock(s) gc131. DOI: 10.1107/S2052252517004262/lc5079gc131sup2.hkl

Structure factors: contains datablock(s) gc140LT. DOI: 10.1107/S2052252517004262/lc5079gc140LTsup3.hkl

Structure factors: contains datablock(s) gc143. DOI: 10.1107/S2052252517004262/lc5079gc143sup4.hkl

Structure factors: contains datablock(s) gc148. DOI: 10.1107/S2052252517004262/lc5079gc148sup5.hkl

Structure factors: contains datablock(s) gc154. DOI: 10.1107/S2052252517004262/lc5079gc154sup6.hkl

Structure factors: contains datablock(s) gc162. DOI: 10.1107/S2052252517004262/lc5079gc162sup7.hkl

Tables of structural data and calculated atomic coordinations, and structural figures. DOI: 10.1107/S2052252517004262/lc5079sup8.pdf

CCDC references: 1532402, 1532403, 1532404, 1532405, 1532406, 1532407
