# Supplementary material for: Halogen bonding in hypervalent iodine and bromine derivatives: halonium salts
Source: IUCrJ. 2017 May 10;4(Pt 4):411–9. doi: 10.1107/S2052252517004262 (PMC5571804; doi:10.1107/S2052252517004262)
Supplement: Supplementary file 8 [file m-04-00411-sup8.pdf]

IUCrJ

Volume 4 (2017)

Supporting information for article:

Halogen bonding in hypervalent iodine and bromine derivatives: Halonium salts

Gabriella Cavallo, Jane Murray, Peter Politzer, Tullio Pilati, Maurizio Ursini and Giuseppe Resnati

Table S1 Crystallographic data and structure refinement parameters for diphenyliodonium perchlorate (**1·ClO$_4^-$**), diphenyliodonium hexafluorophosphate (**1·PF$_4^-$**), and dibenzo[b,d]iodolium chloride (**2·Cl$^-$**).

**Structure**	**1·ClO$_4^-$**	**1·PF$_6^-$**	**2·Cl$^-$**
Formula	$C_{12}H_{10}ClIO_4$	$C_{12}H_{10}F_6IP$	$C_{33}H_{22}Cl_2F_{18}I_2O_3$
Molecular formula	$C_{12}H10I^+ ClO_4^-$	$C_{12}H10I^+ PF_6^-$	$2(C_{12}H_8I)^+$, $3(C_3H_2F_6O)$, $2Cl^-$
Fw	380.55	426.07	1133.21
Crystallographic System	monoclinic	monoclinic	triclinic
Space group	$P2_1/c$	$P2_1/n$	P-1
a (Å)	12.2320(12)	5.9721(5)	11.7183(10)
b (Å)	12.7073(15)	12.9442(12)	13.4322(12)
c (Å)	17.150(2)	18.387(2)	14.8978(13
α (°)	90	90	113.429(11)
β (°)	103.500(8)	96.195(12)	107.498(10)
γ (°)	90	90	101.366(10)
V (Å^3)	2592.1(5)	1413.1(2)	1913.0(3)
T (K)	103	103	103
Z	8	4	2
ρ (g.cm^{-3})	1.950	2.003	1.967
μ (mm^{-1})	2.680	2.432	1.906
Absorption correction T_{min},T_{max}	0.4204, 0.5159	0.3579, 0.4415	0.5999, 0.7458
Collected, independent, obs. [$I > \sigma(I)$] reflections	162962, 18783, 15302	77147, 14805, 12120	49282, 15166, 11243
R_{int}	0.0326	0.0235	0.0357
θ_{max}	43.11	50.00	35.51
Parameters, restraints	405, 190	211, 0	536, 0
$wR(F^2)$, $R[F^2 > 2\sigma(F^2)]$	0.0538, 0.0227	0.0629, 0.0242	0.0621, 0.0309
Goodness of fit	1.064	1.030	1.014
$\Delta\rho$ (min,max)	-1.3, 2.09	-1.21, 1.48	-0.71, 0.73
CCDC number	1532402	1532403	1532404

Table S2 Crystallographic data and structure refinement parameters for phenyl-2-carbomethoxyphenyl-bromonium tetrafluoroborate (**3·BF$_4^-$**), di-*p*-fluorophenylbromonium di-bromide/tetrafluoroborate (**4$_3$·(Br$^-$)$_2$·BF$_4^-$**), and *p*-fluorophenylbromonium dichloride/tetrafluoro-borate (**4$_3$·(Cl$^-$)$_2$·BF$_4^-$**).

**Structure**	**3·BF$_4^-$**	**4$_3$·(Br$^-$)$_2$·BF$_4^-$**	**4$_3$·(Cl$^-$)$_2$·BF$_4^-$**
Formula	$C_{14}H_{12}BBrF_4O_2$	$C_{36}H_{24}B_{0.50}Br_{5.50}F_8$	$C_{36}H_{24}BBr_3Cl_3F_{10}$
Molecular formula	$(C_{14}H_{12}BrO_2)^+$, BF_4^-	$3(C_{12}H_8BrF_2)^+$, 2.5Br$^-$, 0.5BF_4^-	$3(C_{12}H_8BrF_2)^+$, 2Cl$^-$, BF_4^-
Fw	378.9	1053.46	967.99
Crystallographic System	triclinic	cubic	cubic
Space group	*P*-1	$P4_332$	$P4_132$
a (Å)	8.1574(11)	15.4248(7)	15.2905(8)
b (Å)	10.0848(14)	15.4248(7)	15.2905(8)
c (Å)	10.1570(16)	15.4248(7)	15.2905(8)
α (°)	89.158(5)	90	90
β (°)	72.819(4)	90	90
γ (°)	66.607(5)	90	90
V (Å^3)	727.64(18)	3669.9(3)	3574.9(3)
T (K)	103	103	103
Z	2	4	4
ρ (g.cm^{-3})	1.730	1.907	1.799
μ (mm^{-1})	2.871	6.087	3.615
Absorption correction $T_{\min}$,$T_{\max}$	0.4633, 0.5373	0.3440, 0.3991	0.6466, 0.7472
Collected, independent, obs. [$I > \sigma(I)$] reflections	1097, 5592, 4528	13923, 1462, 1253	30649, 2890, 2467
R_{int}	0.0299	0.0512	0.0413
$\theta_{\max}$	34.7	28.47	36.69
Parameters, restraints	200, 0	99, 48	99, 34
$wR(F^2)$, $R[F^2 > 2\sigma(F^2)]$	0.0745, 0.0338	0.0540, 0.0297	0.0517, 0.0246
Goodness of fit	1.020	1.107	1.011
$\Delta\rho$ (min,max)	-0.48, 0.69	-0.42, 0.58	-0.27, 0.51
CCDC number	1532407	1532405	1532406

$ The BF_4^- anion was a mixture of BF_4^- and Br^- with a refined population factor ratio 0.54/0.46.

Table S3 M06-2X/6-311G(d) optimized coordinates of diphenyl iodonium cation **1**.

Center Number	Atomic Number	Atomic Type	Coordinates (Angstroms) X	Y	Z
1	6	0	2.117551	0.410679	1.225303
2	6	0	1.623044	-0.015543	-0.000006
3	6	0	2.117535	0.410756	-1.225295
4	6	0	3.166597	1.324023	-1.209100
5	6	0	3.685419	1.776748	0.000037
6	6	0	3.166612	1.323947	1.209152
7	1	0	1.713806	0.048763	2.162779
8	1	0	1.713779	0.048898	-2.162789
9	1	0	3.576899	1.676753	-2.147775
10	1	0	4.505224	2.485080	0.000054
11	1	0	3.576927	1.676617	2.147844
12	6	0	-1.623044	-0.015543	-0.000006
13	6	0	-2.117551	0.410679	1.225303
14	6	0	-2.117535	0.410756	-1.225295
15	6	0	-3.166613	1.323947	1.209152
16	1	0	-1.713806	0.048763	2.162779
17	6	0	-3.166596	1.324024	-1.209100
18	1	0	-1.713779	0.048899	-2.162790
19	6	0	-3.685419	1.776748	0.000037
20	1	0	-3.576927	1.676617	2.147843
21	1	0	-3.576899	1.676753	-2.147775
22	1	0	-4.505224	2.485080	0.000054
23	53	0	0.000000	-1.386541	-0.000039

Table S4 M06-2X/6-311G(d) optimized coordinates of dibenzo[b,d]iodolium cation **2**.

Center Number	Atomic Number	Atomic Type	Coordinates (Angstroms) X	Y	Z
1	6	0	-1.551948	1.972267	0.000008
2	6	0	-0.733780	0.838246	0.000001
3	6	0	-1.396403	-0.390209	-0.000008
4	6	0	-2.765697	-0.565245	-0.000010
5	6	0	-3.539925	0.591166	-0.000002
6	6	0	-2.933328	1.845436	0.000006
7	1	0	-1.103028	2.958528	0.000016
8	1	0	-3.226880	-1.544728	-0.000017
9	1	0	-4.619543	0.504176	-0.000005
10	1	0	-3.548334	2.737053	0.000012
11	6	0	0.733676	0.838295	0.000001
12	6	0	1.396370	-0.390125	-0.000005
13	6	0	1.551771	1.972367	0.000009
14	6	0	2.765678	-0.565068	-0.000006
15	6	0	2.933158	1.845623	0.000008
16	1	0	1.102788	2.958599	0.000014
17	6	0	3.539833	0.591390	0.000000
18	1	0	3.226925	-1.544521	-0.000012
19	1	0	3.548108	2.737278	0.000012
20	1	0	4.619456	0.504467	-0.000002
21	53	0	0.000041	-1.985244	-0.000018

Table S5 M06-2X/6-311G(d) optimized coordinates of phenyl-2-carbomethoxyphenyl-bromonium cation **3**.

Center Number	Atomic Number	Atomic Type	Coordinates (Angstroms) X	Y	Z
1	6	0	-2.199407	1.040597	-1.289508
2	6	0	-1.663241	0.004718	-0.538141
3	6	0	-2.106567	-0.346281	0.728895
4	6	0	-3.149203	0.398110	1.270025
5	6	0	-3.709913	1.447874	0.547651
6	6	0	-3.240950	1.767871	-0.723504
7	1	0	-1.825495	1.273303	-2.279090
8	1	0	-1.661985	-1.169047	1.275369
9	1	0	-3.523241	0.149973	2.255713
10	1	0	-4.524317	2.018815	0.977419
11	1	0	-3.686070	2.581428	-1.283010
12	6	0	1.325284	-0.031450	-0.697704
13	6	0	2.584203	-0.493239	-1.069609
14	6	0	1.116869	1.087284	0.079183
15	6	0	3.685849	0.234101	-0.618455
16	6	0	2.241431	1.789857	0.512266
17	1	0	0.125260	1.422989	0.352836
18	6	0	3.517277	1.367083	0.166494
19	1	0	4.673757	-0.109916	-0.897681
20	1	0	2.102171	2.672836	1.124291
21	1	0	4.383887	1.919325	0.507735
22	35	0	-0.253534	-1.039220	-1.320123
23	6	0	2.747113	-1.712768	-1.914243
24	8	0	1.798441	-2.348724	-2.310825
25	8	0	4.002411	-2.002578	-2.166708
26	6	0	4.229698	-3.171741	-2.979751
27	1	0	5.307200	-3.248659	-3.075724
28	1	0	3.815606	-4.047857	-2.483909
29	1	0	3.756980	-3.038673	-3.951238

Table S6 M06-2X/6-311G(d) optimized coordinates of di-*p*-fluorophenylbromonium cation **4**.

Center Number	Atomic Number	Atomic Type	Coordinates (Angstroms) X	Y	Z
1	6	0	2.003632	0.422063	1.226610
2	6	0	1.502478	0.014078	-0.000072
3	6	0	2.003823	0.422069	-1.226675
4	6	0	3.072148	1.306967	-1.218358
5	6	0	3.583135	1.734633	0.000093
6	6	0	3.071960	1.306959	1.218463
7	1	0	1.590199	0.065074	2.161644
8	1	0	1.590539	0.065081	-2.161774
9	1	0	3.513544	1.665815	-2.139797
10	1	0	3.513214	1.665803	2.139972
11	6	0	-1.502478	0.014078	-0.000072
12	6	0	-2.003632	0.422063	1.226610
13	6	0	-2.003822	0.422070	-1.226675
14	6	0	-3.071960	1.306959	1.218463
15	1	0	-1.590200	0.065074	2.161644
16	6	0	-3.072147	1.306968	-1.218358
17	1	0	-1.590538	0.065082	-2.161774
18	6	0	-3.583135	1.734633	0.000094
19	1	0	-3.513215	1.665802	2.139973
20	1	0	-3.513543	1.665816	-2.139797
21	35	0	0.000000	-1.201386	-0.000191
22	9	0	4.599264	2.579883	0.000174
23	9	0	-4.599264	2.579884	0.000174

Table S7 M M06-2X/6-311G(d) optimized coordinates of ClO_4^- .

Center Number	Atomic Number	Atomic Type	Coordinates (Angstroms) X	Y	Z
1	8	0	2.658838	2.526931	-0.031162
2	8	0	0.403990	3.489093	-0.067815
3	17	0	1.240299	2.317506	0.155535
4	8	0	0.899315	1.282996	-1.142212
5	8	0	0.874836	1.561716	1.345922

Table S8 M06-2X/6-311G(d) optimized coordinates of BF_4^- .

Center Number	Atomic Number	Atomic Type	Coordinates (Angstroms) X	Y	Z
1	9	0	2.214808	3.493821	1.148901
2	9	0	2.214783	1.503865	0.000000
3	9	0	0.338651	2.830526	0.000000
4	9	0	2.214808	3.493821	-1.148902
5	5	0	1.745763	2.830508	0.000000

Table S9 M06-2X/6-311G(d) optimized coordinates of PF_6^- .

Center Number	Atomic Number	Atomic Type	Coordinates (Angstroms) X	Y	Z
1	15	0	-0.762712	2.322034	0.000000
2	9	0	-2.387104	2.322034	0.000000
3	9	0	-0.762712	3.946426	0.000000
4	9	0	-0.762712	2.322034	1.624392
5	9	0	0.861680	2.322034	0.000000
6	9	0	-0.762712	0.697642	0.000000
7	9	0	-0.762712	2.322034	-1.624392

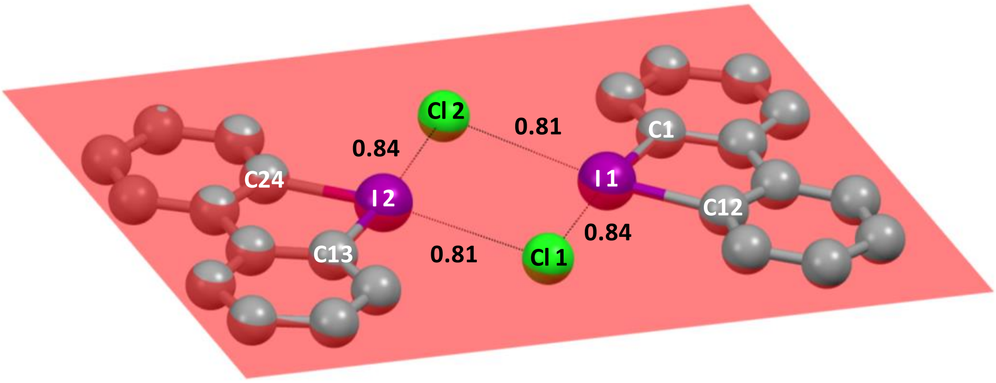

C–I···Cl angles: 172.81° (C1–I1···Cl1) , 171.77° (C12–I1···Cl2)
174.70° (C13–I2···Cl2) , 170.32° (C24–I2···Cl1)

Figure S1 Tetrameric adduct (Mercury 3.8, ball and stick) of the of dibenzo[b,d]iodolium chloride (**2·Cl⁻**). The mean square plane (semitransparent red) through I1, Cl1, I2, and Cl2 evidences that chloride anions enter the elongation of C–I covalent bonds. Hydrogen atoms have been omitted for simplicity; XBs are black dotted lines and respective of Nc values and angles are given close to interactions and at the bottom, respectively. Color codes: Grey, carbon; green, chlorine; purple, iodine.

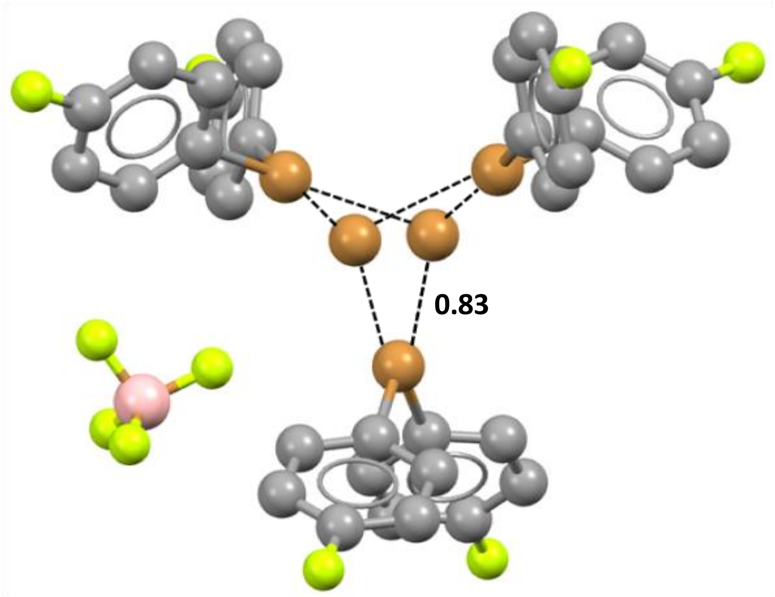

Figure S2 Representation (Mercury 3.8, ball and stick) of the trigonal bipyramidal adduct present in **4_3·(Br^-)$_2$·BF_4^-.** Br···Br^- XBs are black dashed lines and corresponding Nc values are given; hydrogen atoms have been omitted for simplicity. Color code: Grey, carbon; yellowish green, fluorine; brown, bromine; pink, bromine.

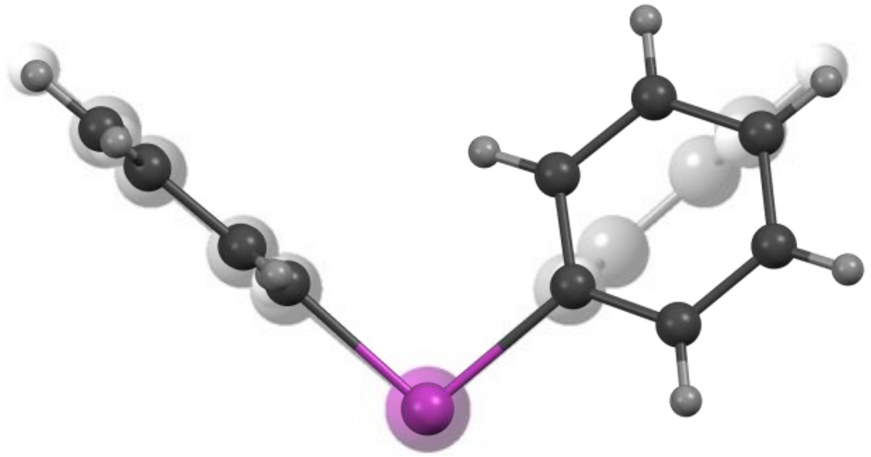

Figure S3 Superposition of the ball and stick structure of diphenyliodonium cation **1** calculated with Gaussian 09 (atoms and bonds with larger sizes and lighter colors in the background) and the X-ray experimental structure (atoms and bonds with smaller sizes and darker colors at the forefront) of the same cation in **1•ClO_4^-**(molecule where the C–I···O angles are 169.52° and 170.97°). Color code: Dark grey, carbon; light grey, hydrogen; purple, iodine.

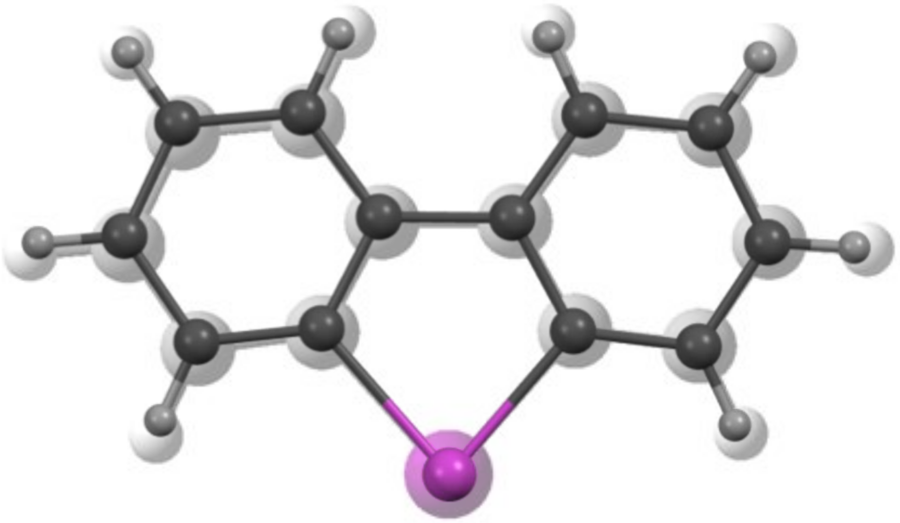

Figure S4 Superposition of the ball and stick structure of dibenzo[b,d]iodonium cation **2** calculated with Gaussian 09 (atoms and bonds with larger sizes and lighter colors in the background) and the X-ray experimental structure (atoms and bonds with smaller sizes and darker colors at the forefront) of the same cation in **2•Cl^-**. Color code: Dark grey, carbon; light grey, hydrogen; purple, iodine.

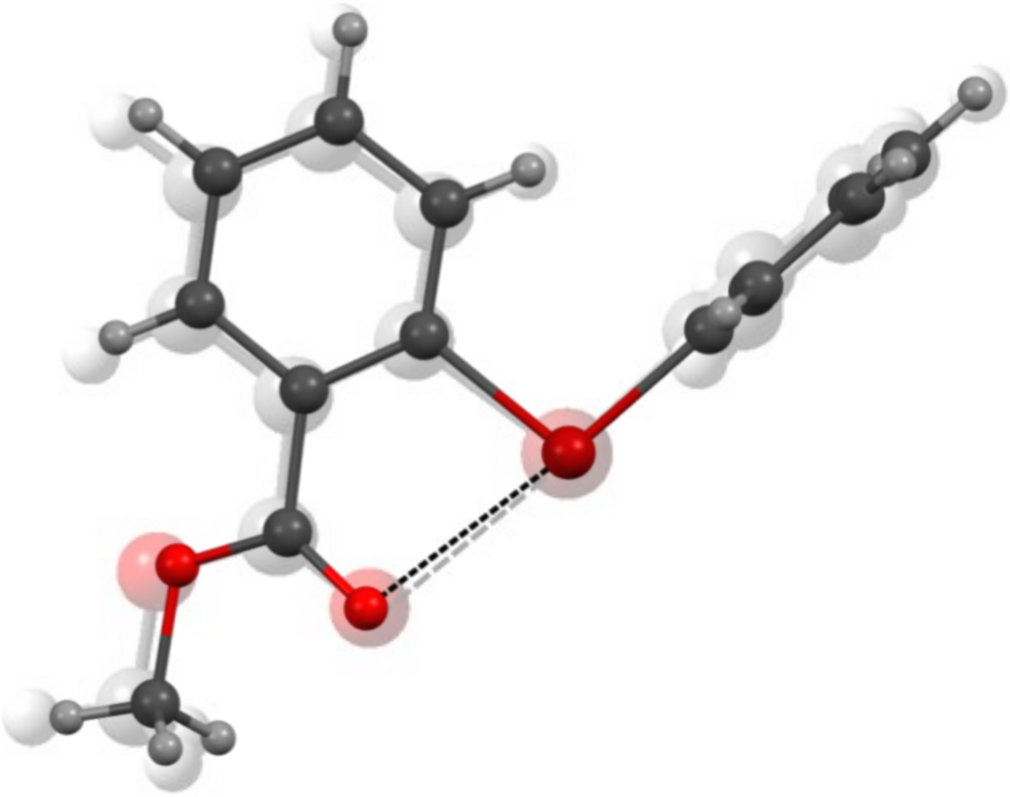

Figure S5 Superposition of the ball and stick structure of phenyl-2-methoxycarbonylphenylbromonium cation **3** calculated with Gaussian 09 (atoms and bonds with larger sizes and lighter colors in the background) and the X-ray experimental structure (atoms and bonds with smaller sizes and darker colors at the forefront) of the same cation in **3•BF$_4^-$**. Color code: Dark grey, carbon; light grey, hydrogen; brownish, bromine; red, oxygen.

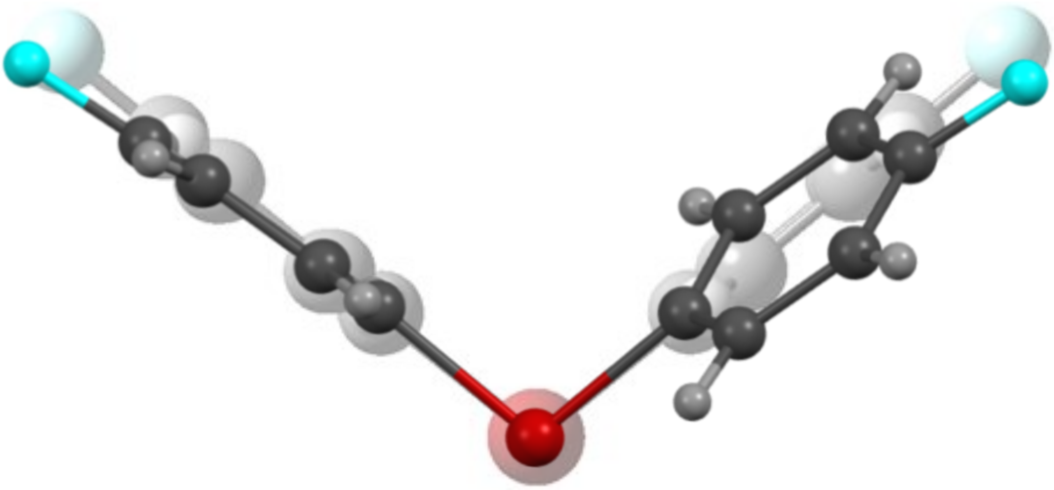

Figure S6 Superposition of the ball and stick structure of di-*p*-fluorophenylbromonium cation **3** calculated with Gaussian 09 (atoms and bonds with larger sizes and lighter colors in the background) and the X-ray experimental structure (atoms and bonds with smaller sizes and darker colors at the forefront) of the same cation in $\mathbf{4_3{\cdot}(Cl^-)_2{\cdot}BF_4^-}$. Color code: Dark grey, carbon; light grey, hydrogen; brownish, bromine; sky blue, fluorine.
